# Association of Charlson Comorbidity Index and ASA Score with Postoperative Mobility in Geriatric Hip Fracture Patients

**DOI:** 10.3390/jcm15062296

**Published:** 2026-03-17

**Authors:** Florian Pachmann, Alexander M. Keppler, Jakob Hofmann, Salome Hagelstein, Christopher Lampert, Carl Neuerburg, Wolfgang Böcker, Leon M. Faust

**Affiliations:** 1 Department of Orthopaedics and Trauma Surgery, Musculoskeletal University Center Munich (MUM), LMU Klinikum, 81377 Munich, Germany; florian.pachmann@med.uni-muenchen.de (F.P.); alexander.keppler@med.uni-muenchen.de (A.M.K.); jakob.hofmann@med.uni-muenchen.de (J.H.); salome.hagelstein@med.uni-muenchen.de (S.H.); christopher.lampert@med.uni-muenchen.de (C.L.); carl.neuerburg@med.uni-muenchen.de (C.N.); leon.faust@med.uni-muenchen.de (L.M.F.); 2 Department of Orthopaedics and Traumatology, Medical Faculty and University Hospital, 40225 Duesseldorf, Germany

**Keywords:** proximal femoral fracture, weight bearing, gait analysis, wearable insole sensors, Barthel Index, ASA, Charlson Comorbidity Index

## Abstract

**Background**: Early mobilization with permission for full weight bearing is a cornerstone of postoperative care after proximal femoral fractures (PFFs). However, its biomechanical implementation during gait remains unclear. Clinical scores such as the Charlson Comorbidity Index (CCI) and the American Society of Anesthesiologists (ASA) classification describe comorbidity burden, but their relationship with actual weight bearing and functional outcome regarding activities associated with daily living is insufficiently understood. **Methods**: In this prospective cohort study, patients aged > 65 years treated surgically for femoral neck fractures (FNFs) or trochanteric femoral fractures (TFFs) were included. Postoperative weight bearing was assessed after 4 to 7 days using sensor-based insoles. Average peak force of the operated limb, normalized to body weight, was the primary outcome. Associations with postoperative weight bearing and functional outcome were analyzed using multivariable linear regression models. **Results**: Early postoperative weight bearing remained below recommended levels, with lower limb loading in TFFs. Higher CCI values were associated with increased loading in TFF patients, and higher ASA classifications with reduced loading. Higher postoperative Barthel Index (BI) was independently associated with increased limb loading. Postoperative BI was influenced by age, preoperative BI, and fracture type. **Conclusions**: Despite permission for full weight bearing, early postoperative limb loading after PFF remains below recommended levels, particularly in TFFs. CCI and ASA show fracture type-specific associations with actual weight bearing, whereas BI is independent of ASA and CCI. The BI may serve as a surrogate parameter to identify patients at risk of insufficient limb loading who may benefit from targeted physiotherapeutic interventions.

## 1. Introduction

Proximal femur fractures (PFFs) are among the most common fractures in older patients and predominantly occur as a result of low-energy falls [1]. These injuries are associated with substantial functional decline, impaired mobility, and, in many cases, a partial or permanent loss of independence in activities of daily living [2]. The vast majority of PFFs comprise trochanteric femoral fractures (TFFs) and femoral neck fractures (FNFs) [3].

The surgical management of PFFs depends on fracture morphology. FNFs in older patients are most commonly treated with endoprosthetic procedures, typically using bipolar hemiarthroplasty and less frequently, total hip arthroplasty depending on age, comorbidities and functional demand. In contrast, TFFs are usually managed with intramedullary nail osteosynthesis. Despite differing surgical approaches, both treatment strategies aim to achieve sufficient primary stability to permit immediate postoperative weight bearing of the affected limb.

In the geriatric population typically affected by PFFs, early mobilization is regarded as a key element of postoperative care, as it has been shown to reduce morbidity and mortality [4]. Consequently, early full weight bearing is widely encouraged. However, it remains unclear to what extent the recommended early mobilization is actually accompanied by adequate biomechanical loading of the operated extremity. In routine clinical practice, mobilization success is predominantly assessed using temporal or functional parameters, such as walking distance, use of walking aids, or the achievement of predefined mobilization milestones. These measures are largely qualitative or semiquantitative in nature and therefore provide only limited information regarding the actual forces acting on the operated limb during gait. Accordingly, the clinical assessment of gait patterns remains largely subjective [5]. The use of wearable devices facilitates analysis and monitoring of postoperative mobility. Sensor-based insoles were used for gait analysis in multiple previous studies in orthogeriatric patients with PFFs and allows for recognition of extremity weight bearing [6,7].

To account for patient-related heterogeneity in postoperative recovery, several clinical and functional scores can be used to characterize baseline health status and functional capacity. The Charlson Comorbidity Index (CCI) and the American Society of Anesthesiologists (ASA) classification allow a structured assessment of comorbidity burden and functional independence. Previous studies have demonstrated associations between these measures and postoperative mobility and physical activity following PFFs [8,9,10,11,12,13]. Both the ASA and CCI have been linked to mortality in patients with proximal femoral fractures, with inconsistent results reported regarding their comparative predictive performance [14,15]. An observational study reported an association between CCI and the postoperative independent walking ability in patients with PFFs [16]. A CCI ≥ 6 was shown to be a predictor of reduced functional outcomes one year after hip fracture surgery [17]. The association of ASA and CCI with postoperative weight bearing and functional status in the early mobilization phase after surgery for hip fracture has not yet been sufficiently investigated.

Therefore, the aim of this study was to assess the relationship between comorbidity burden (ASA, CCI) and the actual postoperative weight bearing of the affected extremity, measured by wearable insoles, as well as functional outcome (Barthel Index, BI) during the early postoperative stage in patients treated surgically for PFFs.

## 2. Materials and Methods

### 2.1. Study Setup and Patient Selection

This prospective observational cohort study was conducted at a Level 1 trauma center in Germany. Data collection was performed following approval by the local ethics committee and took place between February 2020 and December 2025.

For a statistical power of 80% and a significance level set at *p* < 0.05, a minimum of 34 patients per fracture group was required to detect clinically relevant differences in postoperative weight bearing between FNFs and TFFs. The final study population substantially exceeded this requirement, with more than 90 patients included in each fracture group. In addition, the overall sample size of more than 200 patients was sufficient for all applied multivariable regression analyses, fulfilling established requirements regarding the number of observations per predictor variable and thereby providing adequate statistical power for both main effects and interaction analyses.

All subjects provided written informed consent prior to inclusion. The study was conducted in accordance with the Declaration of Helsinki. All patients aged > 65 years who were treated operatively for a FNF or TFF were eligible for inclusion. Exclusion criteria comprised the presence of concomitant fractures, presence of mobility-limiting or gait-altering comorbidities, dementia and cognitive impairment (Mini-Mental State Exam, MMSE ≤ 26). Patients were required to be able to mobilize with or without a walking aid.

Patients with FNFs were treated surgically with either hemiarthroplasty using a bipolar prosthesis or total hip arthroplasty, depending on age, functional status, and pre-existing hip pathology. An Aesculap^®^ CoreHip^®^ System (B. Braun SE, Melsungen, Germany) was implanted in arthroplasty cases. None of the patients with FNFs underwent osteosynthetic fracture fixation. Patients with TFFs underwent fracture fixation using intramedullary nail osteosynthesis (implant: TFN-ADVANCED™ Proximal Femoral Nailing System (TFNA), DePuy Synthes, Raynham, MA, USA). The procedure was performed within 24 h after admission according to standard surgical techniques, ensuring adequate implant positioning or fracture reduction and stable fixation under fluoroscopic guidance. Postoperative treatment took place on our orthogeriatric ward in a multidisciplinary approach together with geriatricians, physiotherapists and nurses according to standardized treatment protocols.

### 2.2. Outcome Parameters

All patients underwent a standardized gait analysis with a wearable device. The required walking distance was 10 m back and forth on a flat plain on the surgical ward without personal assistance. Gait was analyzed using a wireless insole sensor system (Loadsol^®^, Novel GmbH, Munich, Germany). The sensors were fitted into shoes matching the appropriate shoe size of each participant. The insole sensors covered the entire plantar surface of the foot and recorded plantar forces at a sampling frequency of up to 200 Hz. All sensors communicated wirelessly via Bluetooth with a tablet computer (iPad; Apple Inc., Cupertino, CA, USA) which allowed data collection and further analysis. The primary biomechanical outcome was the average peak force (Avg. Pf) applied to the operated limb during gait. Peak force values were extracted for each step, and the mean peak force across all valid steps was calculated. To allow interindividual comparison, forces were normalized to total body weight and expressed as a percentage of body weight (Newton). The Loadsol system has been previously validated and described as a reliable tool for wireless measurement of ground reaction forces [18]. Measurements were performed between postoperative day 4 and day 7. All patients received a standardized pain medication regimen according to World Health Organization (WHO) treatment guidelines. Gait analysis was performed only in clinically stable patients without acute postoperative complications requiring intensive monitoring. Standardized physiotherapy had been initiated prior to measurement in all cases. Although laboratory parameter levels were not incorporated into the analysis, perioperative management followed institutional orthogeriatric protocols aimed at early stabilization and mobilization. No local pain catheter was in use during gait analysis. Patients were allowed to use walking aids of their choice during the measurements. Physiotherapy-guided postoperative mobilization had taken place before our measurements in all cases.

BI was used as a secondary outcome parameter for assessment of perioperative functional status and activities of daily living (ADL). It contains ten items including feeding, personal hygiene, mobility, continence, and transfers. Scores range from 0 to 100, with higher values indicating greater independence and functional ability.

To investigate the influence of comorbidities on postoperative function and mobility after hip fracture surgery, both the CCI and the ASA score were collected for all patients included. The ASA score was obtained from preoperative anesthesiologic examination and documentation. It is based on a comprehensive clinical evaluation including medical history, physical examination, and assessment of previous diseases. It categorizes patients from ASA I (healthy) to ASA VI (brain-dead donor), with higher classes indicating increased perioperative risk. CCI was calculated based on age and weighted scores to predefined chronic conditions, with higher total scores indicating increased mortality and poorer prognosis.

### 2.3. Statistical Analysis

After anonymization, data were exported from REDCap (Research Electronic Data Capture, Vanderbilt University, Nashville, TN, USA) and prepared using Microsoft Excel (MS Office 2016, Microsoft Corp., Redmond, WA, USA) before statistical analysis with IBM SPSS Statistics (Version 29, IBM Corp., Armonk, NY, USA). Normality of data distribution was assessed using the Shapiro–Wilk and Kolmogorov–Smirnov tests. Group comparisons were performed using independent samples t-tests for normally distributed continuous variables and Mann–Whitney U tests for non-normally distributed or ordinal data. Categorical variables were analyzed using the chi-square test or Fisher’s exact test, as appropriate. Associations between clinical variables and postoperative weight bearing and functional performance were evaluated using multivariable linear regression models. To examine potential interaction effects, selected continuous predictors were mean-centered prior to model estimation in order to reduce multicollinearity between main effects and interaction terms. Interaction terms were calculated as the product of the respective centered variables and entered into the regression model. Model fit was assessed using the coefficient of determination (R^2^) and adjusted R^2^. Multicollinearity was assessed using tolerance values and variance inflation factors (VIF). Unstandardized regression coefficients (B) are reported with corresponding 95% confidence intervals. Statistical significance was defined as a two-sided *p* value < 0.05.

## 3. Results

A total of 201 orthogeriatric patients who were admitted and surgically treated for a PFF were included in this prospective cohort study. The mean age was 81.4 years (±6.8), and the majority of patients were female (*n* = 151, 75.1%). The mode value of ASA classification was type 3 (n = 125, 62.2%, range 2–4). The median CCI score was 4 (IQR 1, range 2–12). Median BI before admission was 100 (IQR 5) and median postoperative BI was 65 (IQR 20). Table 1 below gives an overview of demographic data and patient characteristics for the whole cohort and each fracture type separately. The surgical treatment of each fracture type can be seen in Table 2.

There was an almost even distribution between patients with FNFs (n = 106, 52.7%) and TFFs (n = 95, 47.3%). Comparison of the two fracture types revealed non-significant differences in age, body mass index (BMI), sex, ASA and CCI scores as well as BI score pre-admission. Postoperative BI and Avg. Pf. both showed significantly higher values in patients with a FNF.

The main analysis was a linear regression model to identify factors associated with the average weight bearing of the operated extremity (Avg. Pf.). Age, sex, BMI, CCI, ASA, BI before admission and after surgery as well as fracture type (FNF vs. TFF) were included as independent variables. The model demonstrated a satisfactory explanatory performance (adjusted R^2^ = 0.316). All predictors showed VIF values ≤ 1.5. Table 3 below displays the results.

Patients with a FNF showed a significantly higher average weight bearing of the operated limb compared with patients with a TFF. Higher postoperative BI scores, female sex and higher CCI values were significantly associated with higher average weight bearing. In contrast, higher body mass index was associated with lower average weight bearing. Age, ASA score and BI before admission showed no significant association with average weight bearing.

To assess effect modification by fracture type, ASA score, CCI, and postoperative BI were mean-centered and included as interaction terms with fracture type in an additional multivariable linear regression model (adjusted R^2^ = 0.322, all VIF values ≤ 2.87). Significant interactions between fracture type and ASA as well as between fracture type and CCI were seen. In patients with TFFs, higher ASA scores were associated with lower average weight bearing of the operated limb (B = −8.26, 95% CI -16.24 to −0.23, *p* = 0.044), whereas higher CCI was significantly associated with increased average weight bearing (B = 3.85, 95% CI 1.43–6.28, *p* = 0.002). No significant interaction was observed between fracture type and postoperative BI.

Further analysis investigated predictors of postoperative functional status measured by BI. Additional multivariable linear regression was performed. Age, sex, BMI, CCI, ASA, BI before admission and fracture type (FNF vs. TFF) were included as independent variables. Results are shown in Table 4.

In this model, age, BI before admission and fracture type were significantly associated with postoperative BI. Higher BI before admission was associated with increased postoperative BI, while higher age and TFF were significantly associated with a lower postoperative BI. Sex, BMI, ASA score and CCI revealed no significant influence on postoperative BI.

## 4. Discussion

The present study aimed to investigate the association of comorbidities, assessed with ASA and CCI, and early postoperative weight bearing on the operated limb and functional status in patients with PFFs. Patients with FNFs and TFFs were evaluated. Comparison of demographic baseline data showed no significant differences between both fracture groups. The main analysis revealed higher average weight bearing of the operated limb in patients with FNFs than in patients with TFFs. Higher CCI was associated with higher average weight bearing. Effect modification of fracture type was examined in an interaction model, which confirmed the association between higher CCI with higher weight bearing in patients with TFFs, and further showed significant association between higher ASA scores with lower postoperative weight bearing in patients with TFFs. While ASA and CCI had no significant effect on postoperative BI in an additional linear regression model, higher age, lower preoperative BI and TFFs were significantly associated with lower postoperative BI.

Postoperative weight bearing of the operated extremity after a PFF was substantially lower than clinically assumed. Despite the general recommendation of early full weight bearing, the average peak force applied to the operated limb reached only approximately two thirds of body weight, indicating a relevant discrepancy between prescribed mobilization strategies and their actual biomechanical implementation during gait. This discrepancy was particularly pronounced in patients with TFFs, who exhibited significantly lower postoperative limb loading compared with patients suffering from FNFs. These findings during the early postoperative phase are consistent with previously reported results obtained using insole-based load measurements, as well as with studies describing inferior functional outcomes four to six months postoperatively, including reduced Elderly Mobility Scale scores, prolonged Timed Up and Go times, and diminished overall mobility in patients with TFFs [7,19]. Earlier studies primarily attributed these differences to higher age and poorer preoperative general health status [20]. In our cohort, however, no relevant baseline differences were observed with regard to age or surrogate parameters of general health status, such as ASA classification or CCI, suggesting that additional factors must contribute to the observed differences in postoperative weight bearing. To the best of our knowledge, the current literature does not state adequate postoperative peak force levels after hip fracture surgery. Kneiss et al. previously reported a significant difference in peak force levels between the injured and healthy limb after hip fracture surgery [21]. Another study found a significant increase in peak forces applied to the operated limb after hip fracture surgery in a geriatric population throughout the acute hospital stay [22]. In the acute postoperative phase, pain and reduced functional reserves may contribute to limited weight bearing of the operated limb. Full weight bearing as tolerated after hip fracture surgery should be promoted, yet loading the operated limb with full body weight appears unrealistic.

One plausible explanatory approach relates to the anatomical localization of the injury. While most FNFs are intracapsular and therefore largely spare the surrounding musculature, TFFs involve the trochanteric region, which comprises numerous muscle insertions and forms a key component of the vastogluteal sling. Disruption of this functional unit may compromise pelvic stability and force transmission during gait. In line with this hypothesis, recent data have demonstrated significantly reduced hip flexion strength in patients with TFFs, supporting a relevant muscular contribution to the impaired postoperative weight bearing observed in this fracture entity [23].

Postoperative functional status, as reflected by the BI after surgery, was independently associated with higher average weight bearing, whereas preoperative functional capacity showed no such relationship. This finding is not entirely unexpected, as mobility in various activities of daily living represents a central component of the BI. However, our results add a novel aspect by demonstrating that even among patients who are generally mobile, lower postoperative BI scores are associated with reduced actual biomechanical loading of the operated limb.

While the magnitude of postoperative weight bearing cannot be reliably estimated based on the preoperative BI, our findings suggest that postoperative BI values may serve as a pragmatic surrogate marker for the actual biomechanical implementation of weight bearing during gait. This relationship may be of clinical relevance, as it allows an indirect estimation of insufficient limb loading without the need for technically demanding and resource-intensive insole-based gait analyses. Consequently, postoperative functional assessment using the BI could support the identification of patients who may benefit from targeted physiotherapeutic interventions aimed at improving effective weight bearing and gait mechanics.

In contrast, commonly used clinical risk scores demonstrated limited ability to reflect real-world weight bearing. Neither age nor ASA classification was significantly associated with postoperative limb loading in the main analysis, while the CCI showed a weak but significant positive association. This finding appears counterintuitive at first glance, as previous studies have reported associations between these scores and postoperative mobility or functional outcome after hip fracture [11,12,13].

These findings highlight a key conceptual distinction between different assessment instruments. Clinical risk scores such as the ASA classification and the CCI are primarily designed to capture perioperative risk, comorbidity burden, and overall physiological reserve, rather than functional gait performance. None of these instruments directly quantify how weight bearing is biomechanically implemented during gait. Consequently, while higher comorbidity or perioperative risk may translate into worse functional outcomes or delayed recovery, this does not necessarily correspond to reduced limb loading during walking, particularly in the early postoperative phase.

The observed positive association between CCI and postoperative limb loading ap-pears counterintuitive and should be interpreted with caution. Although higher comorbidity burden is generally associated with reduced mobility and disability, our gait analysis necessarily included only patients capable of early postoperative ambulation [12,24]. This methodological requirement results in a select, functionally preserved subgroup and may have introduced selection bias by excluding multimorbid patients with severe functional impairment. As a result, only multimorbid individuals with preserved functional capacity were captured, potentially leading to an overrepresentation of patients capable of achieving higher biomechanical limb loading despite substantial comorbidity burden. Consequently, the observed association may not be generalizable to the broader orthogeriatric population. An additional source of potential selection bias arises from the exclusion of patients with relevant cognitive impairment (MMSE ≤ 26) and those unable to participate in early post-operative mobilization. Cognitive impairment and limited mobility are highly prevalent in the orthogeriatric population and are themselves associated with poorer functional outcomes and reduced recovery potential. Consequently, our study population represents a comparatively higher-functioning subgroup of orthogeriatric patients. The associations observed between comorbidity burden and postoperative limb loading may therefore not be directly transferable to patients with severe cognitive impairment or those unable to ambulate in the early postoperative phase. This limitation should be considered when interpreting the external validity of our findings. Another limitation of this study is the fact that data is limited to the early postoperative phase during acute hospital care. As gait analysis was performed between postoperative day four and seven, early postoperative factors such as pain, postoperative anemia, transient muscle weakness, medication effects, or short-term complications may have influenced weight bearing performance. Although all patients were clinically stable and received standardized perioperative care, these acute influences cannot be fully separated from the measured biomechanical outcomes and should be considered when interpreting the results.

Patient-specific factors also influenced postoperative loading behavior. Male sex and higher body mass index were associated with lower average weight bearing of the operated limb. Previous studies have shown that women tend to regain mobility more rapidly than men in the early postoperative phase after hip fracture surgery [25]. In addition, a higher body mass index has consistently been associated with poorer mobility outcomes and reduced functional performance [26]. Beyond its general association with mobility limitations, increased body weight has been shown to induce altered gait patterns characterized by adaptive joint kinematics and modified energy distribution, which ultimately result in reduced force transmission to the proximal joints of the lower extremity [27]. Together, these findings indicate that individual patient characteristics and behavioral factors play an important role in postoperative gait patterns and the biomechanical implementation of weight bearing, beyond what is explained by clinical risk scores and surgical factors alone.

Finally, determinants of postoperative functional recovery differed from those influencing actual weight bearing. While age, preoperative BI and fracture type were significant predictors of postoperative functional independence, these factors did not consistently overlap with predictors of biomechanical limb loading. This divergence indicates that functional scores and objective gait analysis capture distinct and complementary dimensions of postoperative recovery. Patients may achieve functional independence in daily activities while still underloading the operated limb during gait, which would remain undetected by conventional clinical assessments.

## 5. Conclusions

In this study, early postoperative weight bearing and functional recovery after proximal femoral fracture showed fracture type-specific associations with comorbidity burden and perioperative risk. Despite permission for full weight bearing in both groups, neither fracture group achieved the recommended level of full weight bearing during gait, with patients with TFFs consistently exhibiting lower average limb loading than patients with FNFs. While higher Charlson Comorbidity Index values were associated with increased postoperative weight bearing and higher ASA scores with reduced limb loading in patients with TFFs, functional recovery assessed by the BI was not influenced by ASA or CCI but was primarily determined by age, preoperative functional status, and fracture type.

Furthermore, postoperative loading behavior was influenced by patient-specific factors, including male sex and higher body mass index, both of which were associated with reduced weight bearing. These findings confirm that functional scores, clinical risk profiles, and objective gait analysis capture distinct and complementary dimensions of recovery. Importantly, they underline the need for increased clinical awareness that permission for full weight bearing does not necessarily translate into actual full loading during gait. Postoperative functional assessment using the BI may serve as a pragmatic surrogate to identify patients at risk of insufficient limb loading, who may benefit from intensified and targeted physiotherapeutic interventions aimed at achieving effective full weight bearing.

## Figures and Tables

**Table 1:** Patient characteristics.

	Total (n = 201)	FNF (n = 106)	TFF (n = 95)	*p*-Value
Age (years)	81.4 ± 6.8	81.1 ± 6.7	81.7 ± 6.9	0.650
BMI (kg/m^2^)	23.9 ± 4.6	23.9 ± 4.3	23.9 ± 4.9	0.704
Sex				
Female (%)	151 (75.1)	81 (76.4)	70 (73.7)	0.655
ASA (IQR)	3 (1)	3 (1)	3 (1)	0.480
I	-	-	-	
II (%)	70 (34.8)	40 (37.7)	30 (31.6)	
III (%)	125 (62.2)	62 (58.5)	63 (66.3)	
IV (%)	6 (3)	4 (3.8)	2 (2.1)	
V (%)	-	-	-	
CCI (IQR)	4 (1)	4 (1)	4 (1)	0.998
BI pre-admission (IQR)	100 (5)	100 (5)	100 (5)	0.652
BI postoperative (IQR)	65 (20)	65 (16.25)	60 (15)	<0.001
Avg. Pf. (% of BW)	65.9 ± 15.8	72.1 ± 14.1	58.8 ± 14.6	<0.001

FNF: Femoral Neck Fracture, TFF: Trochanteric Femoral Fracture, BMI: Body Mass Index, ASA: American Society of Anesthesiologists, CCI: Charlson Comorbidity Index, BI: Barthel Index, Avg. Pf.: Average Peak Force, IQR: Interquartile Range. Median values shown for ASA, CCI and BI.

**Table 2:** Surgical treatment according to fracture type.

Fracture Type		
**FNF (%)**	106 (52.7)	
	**Type of surgery (%)**	
	THA cemented	18 (17.0)
	THA cementless	29 (27.4)
	HHA cemented	41 (38.7)
	HHA cementless	8 (7.5)
**TFF (%)**	95 (47.3)	
	**Type of surgery (%)**	
	TFNA™ cemented	70 (73.7)
	TFNA™ cementless	25 (26.3)

FNF: Femoral Neck Fracture, TFF: Trochanteric Femoral Fracture, THA: total hip arthroplasty, HHA: hemi-hip arthroplasty.

**Table 3:** Regression analysis: predictors of postoperative weight bearing in patients with proximal femoral fractures.

Predictor	B	95% CI	*p*-Value
Sex (ref. female)	−6.17	−10.42–−1.91	0.005
BMI (kg/m^2^)	−0.42	−0.83–−0.02	0.041
Age	−0.16	−0.47–0.15	0.309
CCI	1.34	0.07–2.61	0.039
ASA	−3.71	−7.70–0.28	0.068
BI pre-admission	−0.04	−0.14–0.05	0.371
BI postoperative	0.34	0.19–0.50	<0.001
Fracture Type (ref. FNF)	−11.25	−15.00–−7.49	<0.001

BMI: Body Mass Index, ASA: American Society of Anesthesiologists, CCI: Charlson Comorbidity Index, BI: Barthel Index. FNF: Femoral Neck Fracture. B: Beta Coefficient, CI: Confidence Interval.

**Table 4:** Regression analysis: predictors of postoperative Barthel Index in patients with proximal femoral fractures.

Predictor	B	95% CI	*p*-Value
Sex (ref. female)	1.882	−2.010–5.774	0.341
BMI (kg/m^2^)	0.090	−0.283–0.463	0.635
Age	−0.441	−0.713–−0.169	0.002
CCI	−0.239	−1.404–0.926	0.686
ASA	−2.570	−6.187–1.048	0.163
BI pre-admission	0.509	0.257–0.761	<0.001
Fracture type (ref. FNF)	−5.133	−8.486–−1.781	0.003

BMI: Body Mass Index, ASA: American Society of Anesthesiologists, CCI: Charlson Comorbidity Index, BI: Barthel Index, FNF: Femoral Neck Fracture, B: Beta Coefficient, CI: Confidence Interval.

## Data Availability

The raw data supporting the conclusions of this article will be made available by the authors on request.
